# Speech impairment in a large sample of patients with multiple system atrophy: effect of gender and language

**DOI:** 10.1007/s00702-025-03016-9

**Published:** 2025-09-23

**Authors:** Tereza Tykalova, Jan Rusz, Jiri Klempir, Maria Teresa Pellecchia

**Affiliations:** 1 Department of Circuit Theory, Faculty of Electrical Engineering, Czech Technical University in Prague, Technicka 2, 160 00 Praha 6, Czech Republic; 2 Department of Neurology and Centre of Clinical Neuroscience, First Faculty of Medicine, Charles University and General University Hospital, Prague, Czech Republic; 3 Center for Neurodegenerative Diseases (CEMAND), Department of Medicine, Surgery and Dentistry Scuola Medica Salernitana, University of Salerno, Salerno, Italy

**Keywords:** Multiple system atrophy, Sex, Gender, Dysarthria, Acoustic analyses.

## Abstract

Understanding the transferability of language and gender-based phenotypic expression of specific acoustic measures is essential for applying digital speech biomarkers in potential future clinical trials. This study aimed to identify possible gender- or language-related differences in speech between men and women with multiple system atrophy (MSA). A total of 42 male and 40 female MSA patients, along with 41 male and 41 female age-matched healthy controls, were recruited from two centres representing two distinct languages: Czech and Italian. A quantitative acoustic assessment was performed using 12 distinct speech dimensions. No significant clinical differences in MSA patients were found between men and women in terms of age, disease duration, motor severity, or dysarthria severity. MSA patients exhibited significantly worse performance compared to controls for voice quality, pitch breaks, frequency and amplitude vocal tremor, slow and irregular sequential motion rates, imprecise consonants, dynamics of articulation, monopitch, excessive loudness variation, articulation rate, and inappropriate silences (*p* < 0.001). Considering the gender-specific patterns, the female MSA patients manifested more impaired voice quality (*p* < 0.05) and more frequent vocal tremor (*p* < 0.05) then male MSA, while male MSA patients showed slower diadochokinetic rate (*p* < 0.01) and higher excessive loudness variability (*p* < 0.01) than female MSA. The impact of language on disease-related changes appears to be minimal for the majority of acoustic parameters considered. Despite some gender differences, our findings demonstrate that speech-based digital biomarkers in MSA offer high discriminatory power while maintaining good consistency across gender and language.

## Introduction

Multiple system atrophy (MSA) is a rare, adult-onset, rapidly progressive, and fatal neurodegenerative disorder characterized by autonomic failure, poor levodopa-responsive parkinsonism, and varying combinations of cerebellar and pyramidal features (Fanciulli et al. 2015). The estimated prevalence of MSA is 25–30 per 100,000 people over the age of 65 (Schrag et al. 1999; de Rijk et al. 1997), with a similar incidence between men and women. Despite the potential influence of biological gender on the phenotypic expression of MSA, only a limited number of studies have examined potential gender-related differences to date (Cao et al. 2013; Chen et al. 2015; Coon et al. 2015, 2019; Cuoco et al. 2020; Poewe et al. 2022; Yamamoto et al. 2014). Generally, no clear gender-dependent differences in disease progression or survival have been identified among individuals with MSA (see review Leys et al. 2024; Raheel et al. 2023). Yet, a study by Cuoco et al. (2020) investigating 55 MSA patient demonstrated that women with MSA had significantly lower performance on global cognitive abilities, language, visuospatial ability, and attention (Cuoco et al. 2020) and experienced greater deterioration than men after one year of follow-up, particularly in motor functions and attention (Cuoco et al. 2020).

Speech impairment is an early and well-recognized clinical manifestation of MSA, occurring in every patient as the disease progresses (Kluin et al. 1996; Muller et at. 2001; Rusz et al. 2019a). Due to a widespread neuronal atrophy, MSA patients typically develop mixed dysarthria, characterized by a combination of hypokinetic, ataxic and spastic components (Kluin et al. 1996; Rusz et al. 2015, 2019a). In particular, previous objective studies have identified slow rate, vocal tremor, inappropriate silences, excess pitch fluctuation, harsh or strained-strangled voice quality, imprecise articulation and monopitch as the most commonly affected speech dimensions in MSA (Rusz et al. 2015, 2019a). Speech is one of the abilities typically influenced by biological gender, due to differences in the size, shape, and thickness of the vocal folds, the length of the vocal tract, hormone levels, or the effects of menopause and the menstrual cycle in women (Simpson 2009). Although we know that we must attribute some differences to biophysical consequences of anatomical and physiological differences between men and women, no studies have been conducted to investigate the impact of gender on speech performance in MSA.

Previous research has highlighted the promising potential of speech-based digital biomarkers for early and accurate diagnosis, tracking disease progression, and monitoring treatment efficacy or medication side effects (Rusz et al. 2024). However, the stability and robustness of specific acoustic features across different languages need to be determined to enhance the utility of digital speech biomarkers for clinical trials or population screenings. Indeed, several studies have been already published (Fahed et al. 2024; Favaro et al. 2023; Kothare et al. 2024; Kovac et al. 2024; Orozco-Arroyave et al. 2016; Rusz et al. 2021a), generally supporting the evidence that the majority of acoustic features are language-independent, showing similar trends across different languages. However, most of these studies focused on patients with hypokinetic dysarthria in Parkinson’s disease (Favaro et al. 2023; Kovac et al. 2024; Orozco-Arroyave et al. 2016; Rusz et al. 2021a), with only one study has examined hyperkinetic dysarthria in Huntington’s disease (Fahed et al. 2024) and another flaccid-spastic dysarthria in amyotrophic lateral sclerosis (Kothare et al. 2024).

The current study aimed to characterize the patterns of mixed dysarthria in a large sample of individuals with MSA, with the goal to identify potential gender-related speech differences between men and women. Since the patients were recruited from two centres representing different native languages (Czech and Italian), an additional aim was to examine the language dependency of the acoustic features used to quantify speech dysfunction in MSA.

## Methods

### Participants

From 2011 to 2020, 82 consecutive patients with a clinical diagnosis of probable MSA were recruited at two centres, including Charles University and General University Hospital (Prague, Czech Republic) and the University of Salerno (Salerno, Italy). The diagnosis was established according to the consensus diagnostic criteria for MSA (Gilman et al. 2008) by an experienced neurologist in movement disorders (J.K. or M.T.P.). In addition, all patients were clinically monitored for at least four years or until they died to exclude alternative diagnoses other than MSA. The number of participants including their age and gender distribution were similar across both centres. In particular, the Czech MSA cohort consists of 42 patients (21 men, 21 women; mean age 61.2, SD 7.5 years), while the Italian MSA group consists of 40 patients (21 men, 19 women; age 63.1, SD 7.1 years). Most of the MSA patients were treated with antiparkinsonian therapy, taking levodopa alone or in combination with dopamine agonists and/or amantadine. All treated patients were investigated in the medication on-state.

During the clinical visit, each MSA patient was scored according to the Natural History of Neuroprotection in Parkinson plus syndromes-Parkinson plus scale (NNIPPS) (Payan et al. 2011), which enables assessment of all clinical symptoms encountered in Parkinsonism that may potentially influence speech production. Disease duration was estimated based on the self-reported occurrence of the first motor manifestations. Dysarthria type and severity were rated by the experienced speech specialist (T.T., J.R.) using audio recordings of the sustained vowel phonation, syllable repetition and monologue. The dysarthria subtype was determined following the criteria outlined by Darley et al. (1969). Compared to the Italian MSA database, the Czech dataset included a higher proportion of patients with the MSA-parkinsonian subtype than with the MSA-cerebellar subtype (*p* < 0.01). No other significant differences in clinical parameters were observed between the Czech and Italian datasets or between male and female patients (Table 1).

**Table 1:** Patient’s clinical and demographic characteristics

	MSA both languages			MSA Czech			MSA Italian			Group differences	
	Male (*n* = 42)	Female (*n* = 40)	All (*n* = 82)	Male (*n* = 21)	Female (*n* = 21)	All (*n* = 42)	Male (*n* = 21)	Female (*n* = 19)	All (*n* = 40)	Male vs. Female (*p*-value)	Czech vs. Italian (*p*-value)
General											
MSA-parkinsonian subtype	55 (*n* = 23)	68 (*n* = 27)	61 (*n* = 50)	67 (*n* = 14)	86 (*n* = 18)	76 (*n* = 32)	43 (*n* = 9)	47 (*n* = 9)	45 (*n* = 18)	0.24	**< 0.01**
Age (years)	60.9 (7.9)	63.4 (6.5)	62.1 (7.3)	59.4 (8.4)	62.9 (6.2)	61.2 (7.5)	62.3 (7.3)	64.0 (6.9)	63.1 (7.1)	0.11	0.23
Self-reported disease duration (years)	3.8 (1.7)	4.1 (1.7)	4.0 (1.7)	3.8 (1.5)	4.5 (1.6)	4.2 (1.6)	3.9 (1.8)	3.6 (1.7)	3.8 (1.7)	0.51	0.24
Dopaminergic therapy	55 (*n* = 23)	65 (*n* = 26)	60 (*n* = 49)	62 (*n* = 13)	67 (*n* = 14)	64 (*n* = 27)	48 (*n* = 10)	63 (*n* = 12)	55 (*n* = 22)	0.35	0.40
NNIPPS											
Total score	75.6 (24.8)	85.8 (31.6)	80.6 (28.6)	75.8 (26.0)	75.1 (21.5)	75.4 (23.5)	75.4 (24.2)	97.2 (36.9)	85.8 (32.4)	0.11	0.11
Mental	7.0 (4.9)	6.5 (3.3)	6.8 (4.2)	6.2 (4.6)	6.4 (3.4)	6.3 (4.0)	7.8 (5.1)	6.7 (3.6)	7.3 (4.5)	0.62	0.29
Speech (motor examination)	1.9 (0.7)	1.7 (0.7)	1.8 (0.7)	2.1 (0.6)	1.7 (0.6)	1.9 (0.6)	1.7 (0.8)	1.8 (0.8)	1.8 (0.8)	0.41	0.44
Bradykinesia	23.2 (10.2)	27.1 (10.2)	25.1 (10.3)	26.8 (9.0)	26.6 (7.9)	26.7 (8.4)	19.8 (10.3)	27.8 (12.5)	23.5 (11.9)	0.09	0.17
Rigidity	4.7 (3.3)	5.0 (4.2)	4.9 (3.7)	5.2 (3.6)	4.6 (3.8)	4.9 (3.7)	4.3 (3.0)	5.6 (4.6)	4.9 (3.8)	0.71	0.98
Tremor	2.6 (2.9)	3.0 (3.8)	2.8 (3.4)	2.1 (2.8)	2.3 (3.7)	2.2 (3.2)	3.1 (3.1)	3.8 (4.0)	3.4 (3.5)	0.61	0.10
Bulbar/pseudobulbar	8.3 (3.3)	8.3 (3.2)	8.3 (3.3)	8.7 (3.6)	7.8 (3.0)	8.2 (3.3)	7.9 (3.1)	9.0 (3.5)	8.4 (3.3)	0.95	0.80
Cerebellar	4.6 (4.8)	5.1 (4.3)	4.8 (4.6)	4.6 (6.3)	3.4 (4.2)	4.0 (5.3)	4.7 (3.0)	7.0 (3.6)	5.7 (3.5)	0.63	0.08
Dysarthria severity											
Mild	36 (*n* = 15)	30 (*n* = 12)	33 (*n* = 27)	29 (*n* = 6)	29 (*n* = 6)	29 (*n* = 12)	43 (*n* = 9)	32 (*n* = 6)	38 (*n* = 15)	0.59	0.39
Moderate	26 (*n* = 11)	40 (*n* = 16)	33 (*n* = 27)	24 (*n* = 5)	38 (*n* = 8)	31 (*n* = 13)	29 (*n* = 6)	42 (*n* = 8)	35 (*n* = 14)	0.19	0.70
Severe	38 (*n* = 16)	30 (*n* = 12)	34 (*n* = 28)	48 (*n* = 10)	33 (*n* = 7)	40 (*n* = 17)	29 (*n* = 6)	26 (*n* = 5)	28 (*n* = 11)	0.45	0.22
Dysarthria type											
Hypokinetic	17 (*n* = 7)	5 (*n* = 2)	11 (*n* = 9)	19 (*n* = 4)	10 (*n* = 2)	14 (*n* = 6)	14 (*n* = 3)	0 (*n* = 0)	8 (*n* = 3)	0.09	0.33
Ataxic	2 (*n* = 1)	5 (*n* = 2)	4 (*n* = 3)	5 (*n* = 1)	10 (*n* = 2)	7 (*n* = 3)	0 (*n* = 0)	0 (*n* = 0)	0 (*n* = 0)	0.53	0.09
Hypokinetic-ataxic	12 (*n* = 5)	10 (*n* = 4)	11 (*n* = 9)	19 (*n* = 4)	10 (*n* = 2)	14 (*n* = 6)	5 (*n* = 1)	11 (*n* = 2)	8 (*n* = 3)	0.79	0.33
Hypokinetic-spastic	43 (*n* = 18)	28 (*n* = 11)	35 (*n* = 29)	33 (*n* = 7)	38 (*n* = 8)	36 (*n* = 15)	52 (*n* = 11)	16 (*n* = 3)	35 (*n* = 14)	0.14	0.94
Ataxic-spastic	2 (*n* = 1)	13 (*n* = 5)	7 (*n* = 6)	0 (*n* = 0)	5 (*n* = 1)	2 (*n* = 1)	5 (*n* = 1)	21 (*n* = 4)	13 (*n* = 5)	0.08	0.08
Hypokinetic-ataxic-spastic	24 (*n* = 10)	40 (*n* = 16)	32 (*n* = 26)	24 (*n* = 5)	28 (*n* = 6)	26 (*n* = 11)	24 (*n* = 5)	53 (*n* = 10)	38 (*n* = 15)	0.12	0.28

Data are reported as the mean (SD) or the percentage (number)

In addition, we recruited an age- and gender-matched healthy control (HC) groups composed of 42 Czech participants (21 men, 21 women; mean age 62.0, SD 8.0 years) and 40 Italian participants (20 men, 20 women; mean age 62.4, SD 9.6 years). None of the HC subjects reported a history of neurological or communication disorders. All participants were native speakers of their respective languages. Each participant provided written, informed consent. The study received approval from an ethical standards committee on human experimentation and has, therefore, been performed in accordance with the ethical standards established in the 1964 Declaration of Helsinki.

### Speech examination

Speech recordings were performed in a quiet room with a low ambient noise level using a head-mounted condenser microphone (Beyerdynamic Opus 55, Heilbronn, Germany) placed close to the cheek approximately 5 cm from the participant’s mouth. Speech signals were sampled at 48 kHz with 16-bit resolution. Each participant was recorded during a single session. All participants were asked to perform three vocal tasks of (i) sustained phonation of the vowel /a/ per one breath for as long and steadily as possible for at least 10 s, (ii) rapid /pa/-/ta/-/ka/ syllable repetition at least seven times per one breath, and (iii) monologue on a self-chosen topic such as family, hobbies or description of current day for approximately 90 s. The sustained phonation and syllable repetition tasks were repeated twice for every participant.

### Speech analyses

Based on the previous description of speech deficits in MSA (Kluin et al. 1996; Rusz et al. 2015), we selected 12 individual acoustic measurements representing 6 speech dimensions to cover different aspects of hypokinetic, ataxic, and spastic dysarthria commonly encountered in MSA. These 12 chosen acoustic parameters represented different aspects of dysarthria and were found to be only moderately correlated (Pearson: ǀrǀ < 0.60 with highest correlation reached between DDK rate and DDK regularity). Specifically, to evaluate phonation we examined *voice quality* by cepstral peak prominence smoothed (CPPS) (Simek et al. 2021) and *pitch breaks* by proportion of subharmonic intervals (PSI) (Hlavnicka et al. 2019) via speaking task of sustained phonation. To investigate vocal tremor, we quantified *frequency vocal tremor* by modulation depth of frequency tremor (MDFT) (Hlavnicka et al. 2020) and *amplitude vocal tremor* by modulation depth of amplitude tremor (MDAT) (Hlavnicka et al. 2020) through sustained phonation task. Considering oral diadochokinesis, we calculated *slow sequential motion rates* by diadochokinetic rate (DDK rate) (Novotny et al. 2014) and *irregular sequential motion rates* by diadochokinetic regularity (DDK regularity) (Novotny et al. 2014) via syllable repetition task. To asses articulation deficits, we analysed *imprecise consonants* by duration of unvoiced stops (DUS) (Hlavnicka et al. 2017) and *dynamics of articulation* by mel frequency cepstral coefficient global (MFCC global) (Illner et al. 2023) via monologue. To define prosody characteristics, we calculated *monopitch* by standard deviation of fundamental frequency contour (F0 SD) (Illner et al. 2020) and *excessive loudness variation* by standard deviation of speech intensity contour (Int SD) (Rusz et al. 2011) using monologue. To examine speech timing, we computed *articulation rate* by net articulation rate (NAR) (Illner et al. 2022) and *inappropriate silences* by duration of pause intervals (DPI) (Hlavnicka et al. 2017) via monologue. The detailed description of all acoustic features is provided in Table 2.

**Table 2:** The list of acoustic measurements

Deviant speech dimension	Acoustic feature	Definition	References
*(speaking task)*			
*Phonation*			
Voice quality	CPPS (dB)	Cepstral peak prominence smoothed, the unfiltered cepstra are smoothed across fixed frame window and the resulting averaged cepstrum is filtered across quefrencies using moving average filter.	(Simek et al. 2021)
*(sustained phonation)*			
Pitch breaks	PSI (%)	Proportion of subharmonic intervals, defined as the ratio of the total duration of subharmonic intervals per total duration of all voiced intervals.	(Hlavnicka et al. 2019)
*(sustained phonation)*			
*Vocal Tremor*			
Frequency vocal tremor	MDFT (semitones)	Modulation depth of frequency tremor, defined as median modulation depth of dominant frequency tremor. The dominant tremor track was determined from the contour of modal fundamental frequency.	(Hlavnicka et al. 2020)
*(sustained phonation)*			
Amplitude vocal tremor	MDAT (%)	Modulation depth of amplitude tremor, defined as median modulation depth of dominant amplitude tremor. The dominant tremor track was determined from the signal envelope within voiced intervals.	(Hlavnicka et al. 2020)
*(sustained phonation)*			
*Oral diadochokinesis*			
Slow sequential motion rates	DDK rate (syll/s)	Diadochokinetic rate, defined as the number of syllable vocalizations per second.	(Novotny et al. 2014)
*(syllable repetition)*			
Irregular sequential motion rates	DDK regularity (ms)	Diadochokinetic regularity, defined as the standard deviation of distances between following syllable nuclei.	(Novotny et al. 2014)
*(syllable repetition)*			
*Articulation*			
Imprecise consonants	DUS (ms)	Duration of unvoiced stops, defined as the length of the entire unvoiced stop measured from initial burst to vowel onset.	(Hlavnicka et al. 2017)
*(monologue)*			
Dynamics of articulation	MFCC global (-)	Mel Frequency Cepstral Coefficient global, defined as a mean of the standard deviation of the first 16 MFCCs (c1-c16). The coefficient c0 representing signal energy is discarded from the calculation.	(Illner et al. 2023)
*(monologue)*			
*Prosody*			
Monopitch	F0 SD (semitones)	Standard deviation of fundamental frequency contour converted to semitone scale.	(Illner et al. 2020)
*(monologue)*			
Excessive loudness variation	Int SD (dB)	Standard deviation of speech intensity contour extracted from voiced segments.	(Rusz et al. 2011)
*(monologue)*			
*Speech timing*			
Articulation rate	NAR (syll/s)	Net articulation rate, defined as the total number of syllables divided by the total duration of utterance after the removal of pauses.	(Illner et al. 2022)
*(monologue)*			
Inappropriate silences	DPI (ms)	Duration of pause intervals, defined as the median length of pause intervals.	(Hlavnicka et al. 2017)
*(monologue)*			

### Statistical analyses

To enhance speech assessment stability, the values extracted from two repetitions of sustained phonation (syllable repetition) task were averaged for final statistical analyses. To examine language differences, a two-way analysis of variance was conducted for each speech dimension, with LANGUAGE (Czech vs. Italian) and GROUP (MSA vs. HC) treated as between-subject factors. The interaction between GROUP and LANGUAGE was used to assess potential differences in the feasibility of specific acoustic features extracted from one of the studied languages for group separation. A two-way analysis of covariance, with GENDER (male vs. female) and GROUP (MSA vs. HC) treated as between-subject factors and LANGUAGE as a covariate, was applied to explore gender differences in relation to specific acoustic variables. The interaction between GROUP and GENDER was used to determine whether speech performance differences between HC and MSA are more pronounced for a particular gender. Finally, Pearson’s partial correlation analysis, controlling for age and language was performed to test for significant relationships between acoustic and clinical data in the MSA group. Given the exploratory nature of the study, which involved a quantitative analysis of 12 distinct speech aspects, no adjustment for multiple comparisons was performed, and a significance threshold was set at *p* < 0.05. All analyses were performed in MATLAB^®^ (MathWorks, Natick, MA).

## Results

### Language differences

**Fig. 1:**
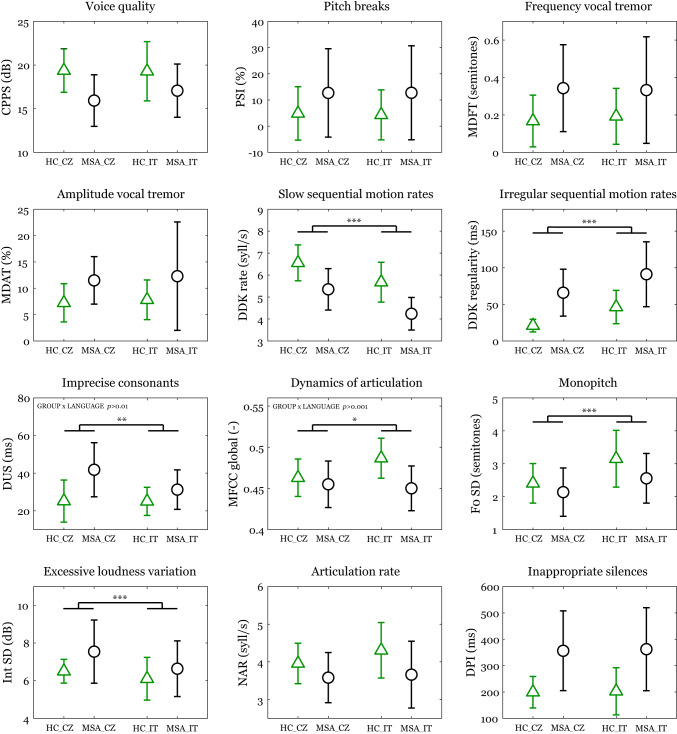
Effect of language on particular acoustic features. Symbols represent mean values and error bars represent SD values. Statistically significant language differences between Czech and Italian participants are depicted by *p < 0.05; **p < 0.01; ***p < 0.001. The group differences between HC and MSA are not depicted as they were significant at *p* < 0.001 for all acoustic features. HC_CZ = Czech healthy controls; HC_IT = Italian healthy controls; MSA_CZ = Czech patients with multiple system atrophy; MSA_IT = Italian patients with multiple system atrophy; CPPS = cepstral peak prominence smoothed; PSI = proportion of subharmonic intervals; MDFT = modulation depth of frequency tremor; MDAT = modulation depth of amplitude tremor; DDK rate = diadochokinetic rate; DDK regularity = diadochokinetic regularity; DUS = duration of unvoiced stops; MFCC global = Mel Frequency Cepstral Coefficient global; F0 SD = standard deviation of fundamental frequency contour; Int SD = standard deviation of speech intensity contour; NAR = net articulation rate; DPI = duration of pause intervals

Based on the significant GROUP × LANGUAGE interactions, the speech dimension of imprecise consonants (*p* = 0.003) was found to be more effective for differentiating MSA in Czech, whereas dynamics of articulation (*p* < 0.001) was more powerful in Italy. The speech performance of MSA patients, compared to HC, was impaired across all 12 speech dimensions (main effect of GROUP, *p* < 0.001) (Fig. 1). When considering LANGUAGE differences independent of disease status, the diadochokinetic rate was slower (*p* < 0.001) and more irregular (*p* < 0.001) in Italy, while the Czech language was characterized by longer consonants (*p* = 0.003), less dynamic articulation (*p* = 0.02) and more monopitch speech (*p* < 0.001) with higher intensity variability (*p* < 0.001).

### Gender differences

**Fig. 2:**
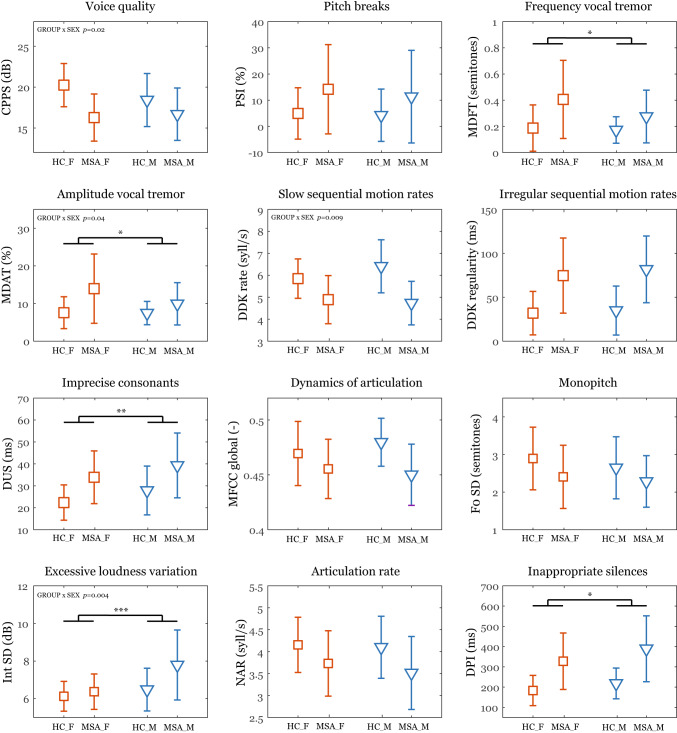
Effect of gender on particular acoustic features. Symbols represent mean values and error bars represent SD values. Statistically significant gender differences between male and female participants are depicted by *p < 0.05; **p < 0.01; ***p < 0.001. The group differences between HC and MSA are not depicted as they were significant at *p* < 0.001 for all acoustic features. HC_F = female healthy controls; HC_M = male healthy controls; MSA_F = female patients with multiple system atrophy; MSA_M = male patients with multiple system atrophy; CPPS = cepstral peak prominence smoothed; PSI = proportion of subharmonic intervals; MDFT = modulation depth of frequency tremor; MDAT = modulation depth of amplitude tremor; DDK rate = diadochokinetic rate; DDK regularity = diadochokinetic regularity; DUS = duration of unvoiced stops; MFCC global = Mel Frequency Cepstral Coefficient global; F0 SD = standard deviation of fundamental frequency contour; Int SD = standard deviation of speech intensity contour; NAR = net articulation rate; DPI = duration of pause intervals

Based on the significant GROUP × GENDER interactions, the female MSA patients manifested more impaired voice quality (*p* = 0.02) and more frequent amplitude vocal tremor (*p* = 0.04) while male MSA patients showed slower diadochokinetic rate (*p* = 0.009) and higher intensity variability (*p* = 0.004). A post-hoc analysis across MSA subtypes revealed a slower DDK rate (t-test: *p* = 0.02) in males with MSA-cerebellar subtype (mean = 4.4, SD = 0.9) compared to males with MSA-parkinsonian subtype (mean = 5.0, SD = 1.0); no significant differences were found between MSA subtypes for intensity variability (*p* = 0.81) in MSA male, voice quality (*p* = 0.22) in MSA female or amplitude vocal tremor (*p* = 0.97) in MSA female patients. In general, MSA patients exhibited poorer speech performance compared to HC across all speech dimensions investigated (main effect of GROUP, *p* < 0.001) (Fig. 2). Considering GENDER differences regardless of disease status, females demonstrated worse speech performance in dimensions reflecting frequency (*p* = 0.04) and amplitude vocal tremor (*p* = 0.03) whereas males exhibited worse performance in imprecise consonants (*p* = 0.002), excessive loudness variation (*p* < 0.001), and inappropriate silences (*p* = 0.01).

### Correlation between acoustic and clinical data

In males, the DDK regularity was positively correlated with NNIPPS total (*r* = 0.45, *p* < 0.05) as well as bulbar (*r* = 0.55, *p* < 0.01) and bradykinesia (*r* = 0.57, *p* < 0.01) subscores (Table 3). In males, the Int SD was also positively correlated with NNIPPS bradykinesia (*r* = 0.41, *p* < 0.05). Additionally, MFCC global was negatively correlated with NNIPPS rigidity (*r* = -0.48, *p* < 0.01) and NAR with NNIPPS total (*r* = -0.42, *p* < 0.05) and NNIPPS bradykinesia (*r* = -0.57, *p* < 0.001) in male population. In females, a negative correlation was observed between the DDK rate and the NNIPPS cerebellar subscore (*r* = -0.56, *p* < 0.01).

**Table 3:** Correlation between acoustic and clinical measurements

Acoustic parameter	Sex	NNIPPS total	NNIPPS Bulbar	NNIPPS Rigidity	NNIPPS Bradykinesia	NNIPPS Cerebellar
CPPS	male	− 0.15	0.02	− 0.35	0.06	− 0.01
	female	− 0.20	− 0.13	− 0.13	− 0.33	0.23
PSI	male	0.00	− 0.11	− 0.05	− 0.05	− 0.12
	female	0.32	0.21	0.28	0.20	0.23
MDFT	male	− 0.02	− 0.04	− 0.08	− 0.08	0.00
	female	0.07	0.16	− 0.04	0.08	0.07
MDAT	male	0.22	0.27	− 0.03	0.25	0.25
	female	0.18	0.22	0.24	0.26	0.16
DDK rate	male	− 0.32	− 0.35	0.19	− 0.38	− 0.33
	female	− 0.29	− 0.05	− 0.10	− 0.29	− 0.56**
DDK regularity	male	0.45*	0.55**	0.06	0.57**	0.23
	female	0.37	0.09	0.35	0.38	0.36
DUS	male	0.11	0.32	0.02	0.04	0.14
	female	0.05	− 0.04	-0.08	0.01	0.08
MFCC global	male	− 0.13	− 0.05	-0.48**	-0.01	0.15
	female	− 0.33	− 0.04	-0.40	-0.28	0.05
FO SD	male	− 0.06	0.10	-0.27	0.00	0.10
	female	− 0.08	0.13	− 0.14	-0.23	0.17
Int SD	male	0.25	0.38	0.05	0.41*	0.22
	female	− 0.04	0.17	− 0.20	0.00	0.12
NAR	male	− 0.42*	− 0.37	− 0.05	− 0.57***	− 0.25
	female	0.03	− 0.06	0.28	− 0.09	− 0.13
DPI	male	0.29	0.22	0.33	0.26	0.11
	female	0.21	0.00	0.34	0.16	0.07

Note: Statistically significant differences between groups: *p < 0.05, **p < 0.01 after Bonferroni’s adjustment for 5 clinical tests performed

*NNIPPS* natural history and neuroprotection on Parkinson plus syndromes-Parkinson plus scale, *CPPS* cepstral peak prominence smoothed, *PSI* proportion of subharmonic intervals, *MDFT* modulation depth of frequency tremor, *MDAT* modulation depth of amplitude tremor, *DDK rate* diadochokinetic rate, *DDK regularity* diadochokinetic regularity, *DUS* duration of unvoiced stops, *MFCC global* Mel Frequency Cepstral Coefficient global, *F0 SD* standard deviation of fundamental frequency contour, *Int SD* standard deviation of speech intensity contour, *NAR* net articulation rate, *DPI* duration of pause intervals

## Discussion

This study provides the first attempt to identify potential gender- and language-specific differences in the speech performance of MSA. We investigated the largest speech sample so far of 82 MSA patients (42 men and 40 women) recruited from two centres representing two distinct languages, Czech and Italian. From a clinical perspective, no significant differences were found between men and woman in age, disease duration, motor severity, or dysarthria severity. In line with previous literature (Kluin et al. 1996; Rusz et al. 2015, 2019a), MSA patients exhibited worse speech performance compared to controls across all 12 speech dimensions investigated including voice quality, pitch breaks, frequency and amplitude vocal tremor, slow and irregular sequential motion rates, imprecise consonants, dynamics of articulation, monopitch, excessive loudness variation, articulation rate, and inappropriate silences. However, gender-specific patterns were revealed only in four speech dimensions. Specifically, the female MSA patients manifested more impaired voice quality and more frequent vocal tremor then male MSA, while male MSA patients showed slower diadochokinetic rate and higher excessive loudness variability than female MSA. The impact of language on disease-related changes seems to be minimal for the majority of acoustic parameters considered, although imprecise consonants was found to be more affected in Czech MSA patients, whereas dynamics of articulation were more impacted in Italian MSA patients. Knowledge about language transferability and gender-phenotypic expression of particular acoustic measures is crucial for the application of digital speech biomarkers in potential future clinical trials involving large-sample datasets or cross-language designs.

Considering gender-related speech difference in parkinsonism, the analysis of gender and speaker’s group interactions revealed poorer voice quality and a higher frequency of vocal tremor in female MSA participants. In agreement with previous study (Rusz et al. 2019a), these laryngeal abnormalities were not influenced by MSA subtype. Interestingly, a previous study (Hertrich et al. 1995) examining 15 patients with advanced stages of Parkinson’s disease reported that female Parkinson’s disease patients had a significantly higher proportion of subharmonic segments, likely due to sexual dimorphism in laryngeal size (Simpson 2009). Although we did not find statistically significant gender-specific differences in the amount of pitch breaks, our female MSA group showed the highest average proportion of subharmonics, at 14.2%, compared to 11.3% in the male MSA group and below 5% in both control groups. Therefore, in general, we may assume that laryngeal abnormalities are more common in MSA female population. In Parkinson’s disease, laryngeal impairment has shown a positive response to dopaminergic therapy, suggesting that it may be attributed to motor dysfunction resulting from the degeneration of dopaminergic pathways in the basal ganglia (Rusz et al. 2021b; Lechien et al. 2018). Therefore, our observation of increased laryngeal abnormalities in females with MSA aligns with previous studies that report greater motor disability in women with MSA over time (Cuoco et al. 2020; Leys et al. 2024).

Contrary, the speech of our MSA male patients were characterised by slower sequential motion rates and more pronounced excessive loudness variations. Indeed, a slow and irregular diadochokinetic rate, along with excessive pitch variations expressed in connected speech, are typical distinctive features of ataxic dysarthria (Darley et al. 1969). A previous study on volumetric correlates found that slow oral diadochokinesis is particularly related to the extent of cerebellar atrophy (Rusz et al. 2019b). One possible explanation for the greater presence of ataxic features in our MSA males compared to MSA females is the higher prevalence of the MSA-cerebellar subtype within the male group. Also, a greater involvement of ataxic features in the MSA male patients is further supported by a revealed negative correlation between diadochokinetic rate and the NNIPPS cerebellar subscore (*r* = -0.42, *p* < 0.001).

Additionally, the other evidence available comes from Parkinson’s disease research, which found no disease status-dependent differences between men and women (Rusz et al. 2022; Houle et al. 2024). However, the study by Rusz et al. (2022) included only de-novo untreated Parkinson’s disease patients, while the study by Houle et al. (2024) focused on mild to moderate Parkinson’s disease patients with an average disease duration of 6 years and no perceptible dysarthria in two-thirds of the database. Indeed, the low severity of dysarthria in previous Parkinson’s disease cohorts (Rusz et al. 2022; Houle et al. 2024) may explain the lack of obvious gender-related speech impairments observed. The advantage of studying speech changes in MSA, compared to Parkinson’s disease, is that all our MSA patients already exhibited dysarthria due to the faster progression of the disease.

All 12 of the acoustic features we investigated proved to be valuable digital biomarkers for MSA as they showed high discrimination power while maintaining strong language robustness. In other words, the expected directions of change due to neurodegeneration were consistent across both the Czech and Italian patient subgroups. These findings are well in accordance with previous studies, (Favaro et al. 2023; Kovac et al. 2024; Orozco-Arroyave et al. 2016; Rusz et al. 2021a) which report largely similar profiles of hypokinetic dysarthria in Parkinson’s disease across different languages. In particular, the majority of acoustic features investigated previously were found to be language-robust, including those that measure the speech dimensions of monopitch (Favaro et al. 2023; Kovac et al. 2024; Rusz et al. 2021a), monoloudness (Rusz et al. 2021a), articulation rate (Favaro et al. 2023; Rusz et al. 2021a), prolonged pauses (Favaro et al. 2023; Kovac et al. 2024; Rusz et al. 2021a), diadochokinetic rate (Kovac et al. 2024; Rusz et al. 2021a), diadochokinetic regularity (Kovac et al. 2024; Rusz et al. 2021a), and voice quality (Kovac et al. 2024; Rusz et al. 2021a).

The analysis of language and speaker’s group interaction revealed a longer duration of stop consonants in Czech MSA, while Italian MSA patients showed increased dynamics of articulation. Since no differences in consonant articulation were observed in the speech performance of Czech and Italian controls, the varying language-dependent duration of consonants in MSA is likely attributable to phonological differences between languages (Haspelmath et al. 2005; Simackova et al. 2012). For instance, Czech is characterized by frequent occurrence of complicated consonant clusters where words may sometimes lack vowels entirely (Simackova et al. 2012). Contrary, the Italian has a predominant consonant–vowel syllable structure with fewer consonant clusters (Haspelmath et al. 2005). Thus, languages containing more complex consonant clusters might be more challenging for MSA patients to pronounce. The lingual-specific differences in MSA dynamics of articulation are more difficult to explain; however, it is well established that MFCC can be language-dependent to some extent, and is even used in language identification systems (Biswas et al. 2023). Indeed, MFCC was developed to mirror human hearing, so it is not surprising that it shows particular sensitivity to a speaker’s style, dialect, or dysarthria subtype.

One potential limitation of our study might be the imbalance in the proportion of MSA subtypes between the Czech and Italian datasets. Specifically, the majority (76%) of Czech MSA patients were classified as having the parkinsonian subtype, compared to only half (46%) of Italian MSA patients. This limitation was partly treated by using language as a covariate in gender-related analyses.

In conclusion, speech-based digital biomarkers in MSA showed high discrimination power while maintaining good gender- and language-consistency. In particular, the revealed gender-specific patterns indicate more common laryngeal abnormalities in MSA female and greater involvement of ataxic features in MSA male population. The impact of language on disease-related changes appears minimal for the majority of biomarkers considered. Future longitudinal studies with more diverse linguistic populations will be helpful to understand prognostic differences between males and females and incorporate gender-related factors in biomarker development and clinical trial design.

## Data Availability

The data that support the findings of this study are available on request from the corresponding author. The data are not publicly available due to privacy or ethical restrictions. Declarations.
